# The impact of hospital presentation time on stroke outcomes: A nationally representative Irish cohort study

**DOI:** 10.1371/journal.pone.0304536

**Published:** 2024-07-12

**Authors:** Elaine Loughlin, Ahmed Gabr, Rose Galvin, Joan McCormack, Olga Brych, Martin J. O’Donnell, Rónán Collins, John Thornton, Joseph Harbison, Margaret O’Connor

**Affiliations:** 1 Department of Ageing and Therapeutics, and Ageing Research Centre, University of Limerick Hospitals Group, Limerick, Ireland; 2 School of Allied Health, Ageing Research Centre, University of Limerick, Limerick, Ireland; 3 National Office of Clinical Audit, Ireland; 4 HRB-Clinical Research Facility, University of Galway, Galway, Ireland; 5 Clinical Lead, National Stroke Programme, Royal College of Physicians of Ireland and Health Service Executive, Ireland; 6 Department of Neuroradiology, Beaumont Hospital, Dublin, Ireland; 7 Department of Medical Gerontology, Trinity College Dublin, Dublin, Ireland; Chinese Academy of Medical Sciences and Peking Union Medical College, CHINA

## Abstract

**Objectives:**

There is conflicting evidence regarding the outcomes of acute stroke patients who present to hospital within normal working hours (‘in-hours’) compared with the ‘out-of-hours’ period. This study aimed to assess the effect of time of stroke presentation on outcomes within the Irish context, to inform national stroke service delivery.

**Materials and methods:**

A secondary analysis of data from the Irish National Audit of Stroke (INAS) from Jan 2016 to Dec 2019 was carried out. Patient and process outcomes were assessed for patients presenting ‘in-hours’ (8:00–17:00 Monday-Friday) compared with ‘out-of-hours’ (all other times).

**Results:**

Data on arrival time were available for 13,996 patients (male 56.2%; mean age 72.5 years), of which 55.7% presented ‘out-of-hours’. In hospital mortality was significantly lower among those admitted ‘in-hours’ (11.3%, n = 534) compared with ‘out-of-hours’ (12.8%, n = 749); (adjusted Odds Ratio (OR) 0.82; 95% Confidence Interval CI [95% CI] 0.72–0.89). Poor functional outcome at discharge (Modified Rankin Scale ≥ 3) was also significantly lower in those presenting ‘in-hours’ (adjusted OR 0.79; 95% CI 0.68–0.91). In patients receiving thrombolysis, mean door to needle time was shorter for ‘in-hours’ presentation at 55.8 mins (n = 562; SD 35.43 mins), compared with ‘out-of-hours’ presentation at 80.5 mins (n = 736; SD 38.55 mins, p < .001).

**Conclusion:**

More than half of stroke patients in Ireland present ‘out-of-hours’ and these presentations are associated with a higher mortality and a lower odds of functional independence at discharge. It is imperative that stroke pathways consider the 24 hour period to ensure the delivery of effective stroke care, and modification of ‘out-of-hours’ stroke care is required to improve overall outcomes.

## Introduction

‘Out-of-hours’ acute hospital admission has been associated with increased morbidity and mortality for patients across a range of medical and surgical conditions—termed the ‘weekend effect’ [1–8]. Several large observational studies have examined this association in acute stroke patients and have found conflicting evidence. An increased mortality has been reported in some studies for those admitted outside of normal working hours [8–16], while other studies have shown no significant difference [17–20]. Weekend admissions have been associated with an increased door to neuroimaging time [12, 14, 21], lower rates of admission to an acute stroke unit [12, 21] and fewer patients returning to their original place of residence [11, 12, 14, 16]- findings which have remained constant even after adjusting for covariates, including age, gender [16] and comorbidities [11]. Differences in study findings may be partially accounted for by differing definitions of the ‘in-hours’ versus ‘out-of-hours’ period, unmeasured confounding, ascertaiment bias and differing study methodologies.

There are several hypotheses as to why stroke care may differ ‘out-of-hours’ compared with ‘in-hours’. ‘Out-of-hours’ process factors such as a greater clinical workload, reduced staffing levels, reduced access to specialist services [8], and reduced access to neuroimaging [22] may be implicated. Unmeasured patient factors may also play a part. Some studies have found that patients presenting ‘out-of-hours’ tend to present with more severe strokes [23]. Much of the existing literature has focused on short-term mortality and has not examined the ‘out-of-hours’ effect in the context of a broad range of patient and process outcomes in acute stroke.

The aim of this study was to evaluate the ‘out-of-hours’ effect on stroke patient and process outcomes within the Irish context.

## Methods

A retrospective cohort study was carried out using anonymized data of consecutive patients admitted to hospital with acute stroke nationally.

### Data availability statement

The data was acquired from the Irish National Audit of Stroke (INAS) database hosted by the National Office of Clinical Audit (NOCA). The INAS database is retrieved from both the national Irish Hospital In-Patient Enquiry (HIPE) system and the national Irish Stroke Portal database. Data is entered at each publicly funded acute stroke receiving hospital site in Ireland into the Stroke Portal and HIPE system by stroke nurses and trained HIPE personnel respectively. INAS is a clinically led, web-based audit managed by the National Office of Clinical Audit (NOCA). This audit is overseen by a governance committee with an independent chairperson, as well as public and patient representation. NOCA is an independent third-party data controller and thus the data used for this study has not been shared directly by the authors. However, anonymized datasets can be openly accessed for research purposes by completing a data request form at https://www.noca.ie/about-noca/access-to-audit-data [24, 25].

Information collected included stroke subtype (ischemic and hemorrhagic), as well as key performance indicators of stroke care. Diagnoses are coded using the International Classification of Disease (ICD), Australian Modification, 10^th^ Edition [26].

Data was shared by INAS for all 25 public hospitals providing acute stroke care in the Republic of Ireland, from January 2016—December 2019. This period was chosen to maximize sample size for analysis.

### Study population

Patients were included if they had been discharged from hospital between the 1st January 2016 and 31st December 2019, aged ≥17 years, and had a principal diagnosis of acute stroke- either ischemic stroke (ICD code I63) or hemorrhagic stroke (ICD code I61). Data on patients with ICD code I64 (‘not specified as hemorrhage or infarction’) and those with an unknown hospital presentation time (‘in-hours’ versus ‘out-of-hours’) were excluded from the analysis. Demographics and baseline characteristics were collected on each patient. Patients who had underwent a mechanical thrombectomy were included, but thrombectomy specific data were not analyzed as part of this study. This population was chosen as it was felt to best reflect contemporary acute stroke care.

### Outcome measures

Outcomes of interest were in-hospital mortality- defined as death during hospital stay- and functional status on discharge, dichotomized into a Modified Rankin Scale (mRS) of 0 to 2 (good functional outcome) and a mRS of 3–5 (poor functional outcome). Modified Rankin Scale of 6 (death) was excluded as these patients were captured in the mortality variable. Process outcomes included door-to-imaging time (mins), door-to-thrombolysis time (mins), thrombolysis rates (%), stroke unit admission (%), length of stay (days) and discharge home rates (%). Outcomes were compared for ‘in-hours’ versus ‘out-of-hours’ periods. Mortality was chosen as a ‘hard’ outcome measure to reduce reporting bias. As outcome measures were recorded at time of death or discharge, there was minimal loss to follow-up. Information bias was reduced by using standardized data collection protocols.

### Time of admission

Patients were categorized by time of admission into ‘in-hours’ and ‘out-of-hours’. ‘In-hours’ was defined as having presented to hospital between 8am-5pm, Monday-Friday. National holidays were included in the ‘out-of-hours’ period. ‘Out-of-hours’ was defined as having presented at any other time outside of the ‘in-hours’ period. These time periods were chosen to reflect standard working hours for medical teams in Ireland.

### Ethics

This study was a retrospective analysis of medical records collected for audit purposes by the National Office of Clinical Audit (NOCA). This audit data is used to inform service delivery and quality improvement nationally and access to, and use of the data for research purposes was approved by NOCA. The ethics committee waived the need for informed consent due to the anonymized nature of the data. This study was approved by the Research Ethics Committee, University Hospital Limerick; Reference: 017/2020, May 2021.

### Statistical analysis

We performed data management using Excel Version 16.43, and data analysis using SPSS Version 28.0.1.1 and R Studio, Version 4.1.2. A descriptive analysis of patient demographics was carried out, categorized by presentation time (‘in-hours’ versus ‘out-of-hours’). The ‘out-of-hours’ category was taken as the reference period in the logistic regression analysis. Continuous data were inspected visually using histograms and P-P plots to assure that they met the assumptions for parametric tests. For continuous variables, comparisons were made using the independent sample t-test (parametric) or Mann-Whitney U test (non-parametric). The Chi-Square test was used for categorical variables.

We performed multivariate logistic regression analysis to determine the association between the independent variables (presentation time, age, gender, good premorbid mRS, stroke subtype, door-to-thrombolysis, thrombolysis administered, acute stroke unit admission, length of stay and door-to-imaging) and both mortality and discharge mRS. Those variables that were significant on the univariate analysis (p < .05) were included within the multivariable logistic regression model. Odds Ratio(s) (OR) with a 95% Confidence Interval (95% CI) were reported. Statistical significance was set at p < .05. Patients were excluded from analyses where missing data points precluded analysis. The Strengthening The Reporting Of Observational Studies In Epidemiology (STROBE) guidelines were adhered to in the reporting of our findings, S1 Checklist [27].

## Results

Data from the INAS database were analyzed from 2016–2019, identifying 14,878 stroke discharges. Patients coded with ICD I61 (ischemic stroke) and I63 (hemorrhagic stroke) were included in the study. Patients coded with ICD I64 (stroke not specified as hemorrhage or infarction, n = 401) were excluded, as were patients with an unknown time of hospital arrival (n = 481), yielding a final dataset of 13,996 patients. Mean age for the overall cohort was 72.5 years, male 56.2%. The percentage of patients under the age 65 years was 26.1% (n = 3649).

‘In-hours’ admissions accounted for 44.3% (n = 6206) of the total stroke patients, with 55.7% (n = 7790) presenting ‘out-of-hours’. There were no differences in age, gender, and pre-morbid functional status (mRS) between those presenting ‘in-hours’ versus ‘out-of-hours’. Intracerebral hemorrhage (ICH) rates were higher during ‘out-of-hours’. Detailed demographics and patient characteristics are shown in Table 1. There was a considerable amount of missing data for the following variables: ‘door to team’ (n = 5184), ‘door-to-imaging’ (n = 1497), pre-stroke Modified Rankin Scale (n = 7218), discharge Modified Rankin Scale (n = 7321) and discharge destination (n = 3407).

**Table 1:** Baseline demographics and characteristics.

Patient Demographics	‘In-hours’ (n = 6206)	‘Out-of-hours’ (n = 7790)	P value
Age (years), mean ± SD*	72.7 ±13.3	72.4 ±13.5	0.21†
Gender: Male, % (n =)	55.4% (n = 3426)	56.8% (n = 4423)	0.08
Pre Stroke mRS 0–2, % (n =)	84.5% (n = 2545)	84.2% (n = 3169)	0.74
Stroke Subtype			.001
Ischemic Stroke, % (n =)	87.5% (n = 5414)	85.6% (n = 6650)	
ICH, % (n =)	12.5% (n = 776)	14.4% (n = 1122)	

*SD: Standard Deviation

† Mann Whitney for non-parametric testing

Process outcomes including door-to-imaging time, door-to-thrombolysis time, and rate of discharge directly to home from hospital were worse ‘out-of-hours’. Length of stay in hospital and admission to stroke unit did not differ significantly by time of presentation. Door-to-imaging time was very long for both time periods. When sub grouped by those who received thrombolysis, this dropped substantially to 32 ± 27.60 mins (n = 560) ‘in-hours’, and 43.1 ± 33.32 mins (n = 730) ‘out-of-hours’ (Table 2). There was a trend towards higher thrombolysis rates for those presenting ‘out-of-hours’ 12.1% vs 10.9%, p = 0.06.

**Table 2:** Outcome measures.

Outcomes	‘In-hours’ (N = 6206)	‘Out-of-hours’ (N = 7790)	P value
**Patient outcomes**			
Overall Mortality, % (n =)	11.3% (n = 534)	12.8% (n = 749)	0.02
Ischemic Stroke	8.5% (n = 348)	8.8% (n = 437)	0.63
ICH*	30.2% (n = 179)	35.7% (n = 308)	0.02
Discharge mRS † (3–5), % (n =) ‡	38.3% (n = 1008)	43.7% (n = 1425)	< .001
Ischemic Stroke	35.7% (n = 840)	41.6% (n = 1204)	< .001
ICH	59.4% (n = 168)	60.5% (n = 221)	.76
**Process Outcomes**			
Door-to-imaging (mins), Mean ± SD§ (n =)	348.9 ± 795.45 (n = 5673)	410.8 ± 739.63 (n = 6826)	< .001
Thrombolysis subgroup			
Door-to-imaging (mins), Mean ± SD§ (n =)	32 ± 27.60 (n = 560)	43.1 ± 33.32 (n = 730)	< .001
Thrombolysis rate, % (n =)	10.9% (n = 594/5414)	12.1% (n = 802/6650)	0.06
Door-to-thrombolysis time (mins), Mean ± SD (n =)	55.8 ± 35.43 (n = 562)	80.5 ± 38.55 (n = 736)	< .001
Stroke Unit Admission, % (n =)	71.6% (n = 4441)	72.2% (n = 5625)	0.38
Ischemic stroke	72.7% (n = 3934)	73.5% (n = 4890)	.283
ICH	63.9% (n = 496)	64.5% (n = 724)	.785
Length Of Stay (days), Mean ± SD (n =)	18.04 ± 29.55 (n = 6206)	18.69 ± 29.62 (n = 7790)	0.582
Discharge Home, % (n =)	57.4% (n = 2700)	50.3% (n = 2949)	< .001

* ICH: Intracerebral Haemorrhage

† mRS: modified Rankin Scale

‡ excluded dead (mRS 6) from analysis

§ SD: Standard Deviation

Univariate and multivariable logistic regression analysis for mortality and discharge mRS is reported in Table 3. Univariate logistic regression identified that ‘out-of-hours’ presentation time, increasing age, female gender, and ICH stroke subtype were each significantly associated with a higher likelihood of both death and poor functional outcome. A good pre-morbid mRS of 0–2 was associated with a significantly lower likelihood of death and higher likelihood of good functional outcome. There was no significant association between either mortality or discharge mRS and door-to-thrombolysis time or thrombolysis administration.

**Table 3:** The effect of patient and process outcomes on in-hospital mortality and discharge mRS (3–5).

Variable	In-hospital Mortality				Poor Functional Outcome (Discharge mRS* 3–5)			
	Univariate		Multi-variable		Univariate		Multi-variable	
	*OR (95%CI)*	*p-value*	*OR (95%CI)*	*p-value*	*OR (95%CI)*	*p-value*	*OR (95%CI)*	*p-value*
Presentation time (‘In-hours’)	0.87 (0.78–0.98)	.025	0.82 (0.68–0.97)	.023	0.80 (0.72–0.89)	< .001	0.79 (0.68–0.91)	.001
Age (years)	1.058 (1.052–1.06)	< .001	1.05 (1.04–1.06)	< .001	1.05 (1.05–1.06)	< .001	1.04 (1.03–1.05)	< .001
Gender (Male)	0.66 (0.59–0.74)	< .001	0.88 (0.74–1.05)	.160	0.60 (0.54–0.66)	< .001	0.89 (0.76–1.03)	.113
Good pre-morbid mRS (0–2)	0.29 (0.25–0.35)	< .001	0.43 (0.35–0.53)	< .001	0.006 (0.003–0.01)	< .001	0.006 (0.003–0.01)	< .001
Stroke Subtype (ICH†)	5.32 (4.67–6.07)	< .001	5.45 (4.50–6.60)	< .001	2.35 (1.99–2.78)	< .001	2.17 (1.73–2.72)	< .001
Door-to-thrombolysis (mins) ‡	1.00 (0.99–1.00)	.523	-	-	1.00 (0.99–1.01)	.589	-	-
Thrombolysis administered‡	1.01 (0.83–1.23)	.927	-	-	1.08 (0.91–1.29)	.376	-	-
Stroke unit admission	0.48 (0.42–0.54)	< .001	0.46 (0.38–0.55)	< .001	1.63 (1.43–1.85)	< .001	1.55 (1.28–1.88)	< .001
Length of stay (days)	0.993 (0.99–0.996)	< .001	0.991 (0.987–0.995)	< .001	1.06 (1.05–1.062)	< .001	1.058 (1.05–1.06)	< .001
Door-to-imaging (mins)	0.999 (0.998–0.9993)	< .001	0.9986 (0.9983–0.9989)	< .001	0.9998 (0.9997–0.9999)	< .001	0.9996 (0.9995–0.9998)	< .001

* mRS: modified Rankin Scale

† ICH: Intracerebral Haemorrhage

‡ Not included within multivariable logistic regression due to non-significance within univariate analysis

Stroke unit admission and increasing length of stay were associated with a significantly lower likelihood of death but a higher likelihood of poor functional outcomes. Longer door-to-imaging time was associated with lower likelihood of both death and poor functional outcomes. Both of these findings must be interpreted with caution due to the observational nature of the data and the fact that they were not our primary outcomes of interest.

Factors associated with an increased odds of mortality on univariate analysis were considered for inclusion in the multivariable logistic regression model. Multivariable logistic regression identified that ‘in-hours’ was associated with both a lower likelihood of death (odds ratio (OR), 0.815 (95% CI 0.68–0.97), p = 0.023) and poor functional outcome (OR, 0.787 (95% CI 0.68–0.91), p < .001) when adjusted for age, gender, length of stay, stroke subtype, door-to-imaging time, pre-stroke mRS and stroke unit admission.

## Discussion

This study demonstrates that a higher proportion of ischemic and hemorrhagic strokes present to hospital outside of normal working hours, which has important implications in terms of healthcare organization and delivery of stroke services. Prior studies on the ‘weekend effect’ in acute stroke care have reported inconsistent results, with some showing admission outside of normal working hours to be associated with a higher mortality [8–12, 28, 29], while other studies have not shown this association [17, 19, 20].

This nationally representative cohort study demonstrated that admission during ‘out-of-hours’ was associated with a higher overall mortality for ICH and a higher likelihood of a poor functional outcome for stroke when compared with admission ‘in-hours’, which persisted after adjustment for available confounding factors, including age, gender, and pre-morbid functional status (mRS).

When assessed based on stroke subtype, mortality was not significantly increased for ischemic stroke, while ICH mortality was 5.5% higher ‘out-of-hours’; a finding consistent with previous literature [29, 30]. Our findings support those of the Stroke Improvement National Audit Programme UK, where inadequate quality of care during ‘out-of-hours’ was highlighted [23]. There are several possible hypotheses as to why ICH mortality is higher ‘out-of-hours’. In our study, there was evidence of organizational factor differences in the ‘out-of-hours’ period with door-to-imaging time and door-to-thrombolysis time being significantly more prolonged. These factors may have contributed to a less standardized approach to ICH management ‘out-of-hours’. Stroke severity measures such as National Institute of Health Stroke Service, Glasgow Coma Scale or ICH-Score were not recorded within the INAS database, therefore it was difficult to extrapolate whether an increased ICH mortality was due to an increased ICH severity ‘out-of-hours’.

Despite the significant reductions in ischemic stroke mortality over the last 20 years [31], mortality for ICH has remained constant [32]. Implementation of a bundle of care has the potential to enhance and streamline the delivery of ICH management both ‘in-hours’ and ‘out-of-hours’ [33]. A secondary analysis of the INTERACT 2 study noted no association between ‘out-of-hours’ admission and ICH related death or disability [34] suggesting a potential protective effect from standardized guideline-based management of ICH employed within a clinical trial setting, which is lacking in routine clinical practice. In a study by Parry-Jones et al., implementing an acute ICH bundle of care incorporating acute anticoagulation reversal, standardized guideline based management of blood pressure lowering, a care pathway for neurosurgical referral and avoidance of palliation within 48 hours was associated with a 44% reduction in 30-day case fatality [35].

A higher chance of a poor functional outcome seen in ischemic stroke for those admitted outside of office hours should be given significant consideration. The fact that time of admission impacts on functional status at discharge must be explained by either process factors or patient factors. Either patients presenting ‘out-of-hours’ represent a higher risk population with a worse prognosis, or peri-admission care has long lasting implications with effects seen even at time of discharge. Interestingly, there was no significant difference in functional outcome seen in those with ICH. This may be perhaps explained by an increased mortality seen in this group, and thus measured only in the mortality variable. Furthermore, those admitted ‘out-of-hours’ had a lower probability of discharge directly home.

There was an association between longer door-to imaging time and better survival and functional outcomes. This is perhaps explained by confounding bias with milder or non-disabling strokes receiving less-urgent imaging. There was a large number of patients with very prolonged imaging times. We hypothesize that these were likely subacute stroke cases, or transient ischemic events, where emergent imaging was not clinically required. Despite this, when sub-grouped by those who received thrombolysis, door-to-imaging time was significantly shorter during normal office hours. This may reflect a more streamlined acute stroke service during ‘in-hours’ run by a dedicated stroke team, with extensive experience in dealing with ‘FAST’ calls.

Furthermore, a trend towards higher rates of thrombolysis ‘out-of-hours’ was noted, despite more prolonged door-to-imaging times. This may represent ‘riskier’ thrombolysis decision making ‘out-of-hours’, carried out by a general on-call service.

The reasons for the differential outcomes between ‘out-of-hours’ and ‘in-hours’ is poorly understood. There may have been unmeasured differences in risk factor profile between those presenting ‘out-of-hours’, such as higher rates of hypertension, diabetes, and other co-morbidities. While we had no data examining the impact of stroke complications on mortality and outcomes, including aspiration pneumonia or urosepsis, it is conceivable that there may have been a delay in diagnosis and management of such complications by on call teams ‘out-of-hours’, as well as less stringent management of important acute factors including blood pressure and blood glucose. While not investigated in our study, it is probable that the lack of allied health professional expertise during the ‘out-of-hours’ period may also have had an impact, particularly in terms of the management of dysphagia. It is conceivable that some patients were denied oral intake for a longer period with others being inadvertently given oral intake in the absence of a dysphagia assessment during the ‘out-of-hours’ period. This data was not available from NOCA for 2016–2019.

While stroke unit admission is known to be protective for stroke outcomes [36], in this study, presentation time did not impact on the likelihood of stroke unit admission. Nevertheless, other unmeasured operational factors such as reduced staffing, competing clinical demands or a lack of specialist stroke expertise ‘out-of-hours’ are likely to have been implicated in the poor functional outcomes and increased mortality seen in those admitted ‘out-of-hours’. Stroke unit admission yielded conflicting results with reduced overall mortality but a higher likelihood of poor functional outcomes observed. The association between stroke unit admission and lower mortality may possibly be explained by early deaths of severe strokes in the emergency department or critical care units before patients would have made it to the stroke unit. The observed increase in poor functional outcomes is likely due to a selection bias, with more severely disabled strokes being prioritized for the limited stroke unit bed capacity within the Irish setting.

A quality improvement process focusing on factors specific to the ‘out-of-hours’ period is essential to identify factors and modify the increased risk associated with presentation outside of normal office hours for stroke patients in Ireland. In particular we propose that this should focus on the acute management of ICH, where an increased mortality risk was seen.

### Strengths and limitations

Our study represents the largest Irish study looking at the differential impact of hospital presentation timing on patient outcomes in acute stroke. As it was a nationally representative study, there is good external validity. Data was captured by trained specialist stroke nurses, using a standardized national audit collection and reporting process. This audit reported on patient level data. Important key performance measures were accurately represented.

The study has several limitations. Firstly, INAS represents a national audit rather than a population-based registry where participation is not mandatory, and some data variables had a large amount of missing data. This has the potential to introduce bias in either direction and thus results should be interpreted with caution. Secondly, stroke severity on admission was not captured precluding assessment for confounding by stroke severity. However, the aim of the study was to explore outcomes for hypothesis generation rather than to inform on causality. Thirdly, only in-hospital mortality was captured by INAS, and thus this may underestimate the true mortality rate. Similarly, discharge mRS may not accurately capture long-term functional status. Fourthly, this study compared ‘in-hours’ with all other times, including nights and weekends, which may have masked some variation in care across the ‘out-of-hours’ period. Lastly, we had limited information on other important confounders such as patient comorbidities including hypertension, diabetes and hypercholesterolemia, acute stroke complications such as aspiration pneumonia and urosepsis and ischemic stroke sub-types.

## Conclusion

Our study found that over 50% of stroke presentations occur outside of normal office hours, with significant implications in terms of morbidity and mortality. This study demonstrates for the first time in an Irish setting the presence of an ‘out-of-hours’ effect in acute stroke with a higher mortality for intracerebral hemorrhage and poorer functional outcomes on discharge for ischemic stroke observed for those presenting during this period. This has important implications for the delivery of national stroke services. Key performance indicators such as door-to-thrombolysis and door-to-imaging times were longer ‘out-of-hours’. There is a need to optimize the care pathways provided by non-specialist medical services outside of normal working hours for acute stroke patients. Ongoing quality improvement identifying barriers and facilitators to timely stroke treatment ‘out-of-hours’ is critical, and the development of a standardized ICH management protocol to streamline treatment care processes during ‘out-of-hours’ is required.

## Supporting information

S1 Checklist STROBE checklist. (DOCX)

## Abbreviations

• mRS: Modified Rankin Scale
• ICH: Intracerebral Hemorrhage
• INAS: Irish National Audit of Stroke
• SD: Standard Deviation
